# Psychometric validation of a patient-reported outcome questionnaire (Qualipsosex) assessing the impact of psoriasis and psoriatic arthritis on patient perception of sexuality

**DOI:** 10.1097/MD.0000000000024168

**Published:** 2021-01-08

**Authors:** Eric Lespessailles, Emmanuel Mahé, Ziad Reguiai, Edouard Begon, François Maccari, Nathalie Beneton, Guillaume Chaby, Carole Rosenberg, Emmanuelle Dernis, Fabienne Roux, Isabelle Henry-Desailly, Bénédicte Charles, Cyril Ferdynus, Marc Marty, Eric Esteve

**Affiliations:** a Department of Rheumatology, Regional Hospital of Orleans; b University of Orleans, EA 4708, I3MTO Laboratory; c Department of Dermatology, Hôpital Victor Dupouy, Argenteuil; d Department of Dermatology, Polyclinique Courlancy-Bezannes, Reims; e Department of Dermatology, Hôpital René Dubos, Pontoise; f Hospital Begin, Saint Mandé; g Department of Dermatology, CH Le Mans; h Department of Dermatology, Hôpital Nord, University of Amiens-Picardie, Amiens; i Department of Rheumatology, Hôpital Victor Dupouy, Argenteuil; j Department of Rheumatology, CH Le Mans; k Department of Rheumatology, Saint Joseph Hospital, Paris; l Service de Rhumatologie CHU Amiens; m France psoriasis, Paris; n CHU La Réunion, Unité de Soutien Méthodologique, F-97400, Saint-Denis, La Réunion; o INSERM, CIC 1410, F-97410, Saint Pierre, La Réunion; p Department of Rheumatology, Henri Mondor Hospital, Creteil; q Department of Dermatology, Regional Hospital of Orleans, Orleans, France.

**Keywords:** psoriasis, psoriatic arthritis, quality of life, questionnaire, sexuality

## Abstract

Psoriasis (Pso) and psoriatic arthritis (PsA) frequently have a negative impact on patients’ sexual health. We have developed a specific questionnaire assessing the impact of Pso and PsA on patient perception of sexuality: the QualipsoSex Questionnaire (QSQ). The aim of the present study was to further validate this questionnaire by checking its psychometric properties including validity, reliability, and responsiveness.

A cross sectional observational study with a longitudinal component for responsiveness and test–retest reliability was performed in 12 centers in France including 7 dermatologists and 5 rheumatologists. Psychometric properties were examined according to the COnsensus-based Standards for the selection of health Measurement INstruments (COSMIN) check-list.

At baseline, 114 patients had Pso and 35 patients had PsA including 17 peripheral arthritis, 4 axial disease, 13 patients with both axial disease and peripheral arthritis and one patient with an undifferentiated phenotype. The mean Pso Area and Severity Index score was 12.5. Genital organs were involved in 44.7% of Pso cases. Internal consistency, construct validity, and reliability were good with Cronbach's α coefficient, measure of sampling adequacy and intraclass correlation coefficient respectively at 0.87, 0.84, and 0.93. The QSQ also demonstrated acceptable sensitivity to change.

The QSQ has demonstrated good psychometric properties fulfilling the validation process relative to the recommendations of the COSMIN check list. The QSQ is simple to score and may hopefully be valuable in clinical practice and in clinical trials.

## 1 Introduction

Psoriasis (Pso) and psoriatic arthritis (PsA) are both heterogeneous and multifaceted diseases.^[1–3]^

Beyond cutaneous and rheumatic conditions both diseases are known to have major psychological and social consequences^[4]^ but may also have a negative impact on a patient's sexual health.^[5,6]^ Psoriasis and PsA may lead to sexual difficulties that should be managed as part of the global patient health status.

Although numerous patient-reported outcomes have been developed and validated in both Pso^[7,8]^ and PsA^[9,10]^ to better answer unmet needs^[11,12]^ and discrepancies between physicians’ and patients’ opinions, there is a lack of a specific questionnaire assessing the impact of Pso and PsA on patient perception of sexuality. We previously published the preliminary development of the QualipsoSex Questionnaire (QSQ).^[13]^ In the article, we described the qualitative step aimed at defining the concept and content of the QSQ in order to generate its dimensions, the questions asked and the framework of responses. The second step, a quantitative one, was then carried out to determine the relevant dimensions and questions.

After this first part of the development of the QSQ which defined the content validity^[13]^ the aim of the present work was to further validate QSQ by checking its psychometric properties including internal consistency, construct validity, criterion validity, reliability and responsiveness,^[14]^ and two cut-offs to interpret the scores.

## 2 Material and methods

### 2.1 Overall organization

A cross sectional observational study with a longitudinal component for responsiveness and reliability was performed in 12 centers in France. Applicable local and general regulations were respected and the project was approved by the ethics committee (CPP-EST-I Dijon on March 2017). Each person solicited for this study had complete freedom to participate or not.

### 2.2 Patients

In each center consecutive adult patients fulfilling the participation criteria were solicited to participate in the study. If the patient agreed to participate, they were invited to an inclusion visit and were then considered as belonging to the population of included patients.

### 2.3 Participation criteria were the following

- Patients should be aged ≥18 years.
- Definite Pso examined in dermatology outpatient clinics in the participating centers with a moderate to severe cutaneous Pso^[15]^ (Psoriasis Area and Severity Index [PASI]^[16]^ score >10/72 or a body surface area >10% or a Dermatology Life Quality Index [DLQI]^[17]^ score >10/30).

And/or an active PsA according to the ClASsification of Psoriatic ARthritis CASPAR group criteria^[18]^ with at least 3 tenderness joints out of 78 and 3 swollen joints out of 76 in the case of peripheral forms of the disease, and with a Bath Ankylosing Spondylitis Disease Activity Index (BASDAI) ≥4 in the case of axial forms of the disease.^[19]^ Both the cutaneous and the articular involvement should have begun at least 6 months earlier.

Patients with a major depression on clinical judgment and with a high “depression” score on the Hospital Anxiety and Depression Scale (e.g., ≥14)^[20]^; pregnant patients and patients with a significant major comorbidity that might significantly alter the patient's sexual quality of life according to the investigator's judgement were excluded.

### 2.4 Data collection

The patients were scored on the Numeric Rating Scale (NRS) of the QSQ; they also completed an NRS on fatigue, the Hamilton Anxiety Depression scale and answered question number 9 in the DLQI adapted to this study: “In the last seven days, has your cutaneous and/or joint problem made your sexual life difficult?” Demographic data and medications were collected. Dermatologists recorded clinical features of Pso including the PASI, the current localization and Body Surface Area (BSA) in percentage. Rheumatologists recorded the clinical phenotypes (axial, peripheral, or both) of the PsA.

Patients with longitudinal assessments (responsiveness and reliability) were asked the following question: “*Do you think that your sexual quality of life has improved since the first visit*?” and had to rate their global assessment of change in their disease since the first visit.

### 2.5 Qualipsosex score

The Qualipsosex is a self-questionnaire based on 10 questions that evaluate the perceived impact from the patient point of view of their Pso with cutaneous and/or articular expression on their sexual quality of life.

Six questions concern the partner's approach (Q1, Q2, and Q4–Q7), one concerns sexual desire (Q3) and three relate to sexual activity (Q8–Q10).

Each question is scored from 0 (not at all) to 4 (always). The total score may vary from 0 (no impact) to 40 (maximum impact). Patients have to answer by circling the number (0, 1, 2, 3, or 4) that best fits their personal experience in the last 3 months.

In addition to these 10 questions, 4 supplementary questions were included but do not contribute to the final score: two questions explore the global impact of the disease on sexual quality of life; one is relative to the cutaneous expression of the disease, while the other is relative to the rheumatic condition. These two questions permit a separate analysis of these two components of the disease on the QSQ score.

One question assesses the patient's expectations about the effect of treatments on their sexual quality of life. This question was meant for future exploratory studies.

One question explores the suffering associated with the lack of interest shown by medical doctors in the sexual quality of life of their patients.

### 2.6 Psychometric properties analyses

Psychometric properties were examined according to the COnsensus-based Standards for the selection of health Measurement INstruments (COSMIN) check-list.^[21]^

The content validity was established in the previous steps of the questionnaire development.^[13]^

To calculate the total score, the scores of the 10 questions were summed. To manage any missing or irrelevant data on the QSQ, it was decided that:

- if an answer to a question was missing, the lowest possible value (i.e., 0) was taken into account for the calculation of the score. If more than 2 answers were missing, it was decided not to calculate the score.
- If several adjacent digits were circled, the average of the 2 digits was taken into account for the calculation of the score.
- If several non-adjacent digits were circled, the data were considered to be missing.
- If a mark was made between 2 digits, the average was taken.

The validity of internal consistency was measured^[22]^ using the Cronbach's α coefficient. Scores of this coefficient between 0.7 and 0.9 are considered as indicating a good internal consistency.

The structural validity of the questionnaire was analysed by an exploratory factorial analysis on the baseline questionnaires. Convergent and discriminant validities were estimated based on the multitrait-multimethod matrix proposed by Campbell and Fiske.^[23]^

Reliability was assessed using the intraclass correlation coefficient (ICC) between the score at baseline and the score at day 7 in patients who had indicated “no change” in their global assessment of change in their disease at day 7.

The criterion validity was assessed using the Pearson's correlation coefficient between the QSQ score at baseline and the adapted score on question 9 of the DLQI at baseline.

Responsiveness was evaluated by the effect size of the score changes between baseline and the 3 to 4 months visit in patients who reported improvement (moderate or high improvement) on a global assessment of their disease. The mean change from baseline to 3 to 6 months after the treatment change divided by the SD of this change was calculated.

Interpretation of the QSQ score was evaluated by the cut-off value for the Patient-Acceptable Symptom State (PASS). This PASS score was estimated as the third quartile of the Qualipsosex score of patients considering themselves in an acceptable state at baseline (i.e., who answered yes at baseline to the question “If your sexual quality of life remains unchanged, would you consider it acceptable?”).

The Minimal Clinically Important Difference (MCID) was calculated by the difference between the mean score of the QSQ of patients claiming an improvement in their sexual quality of life and that of patients claiming not to experience any improvement in their sexual quality of life at 3 to 6 months as compared to the baseline inclusion visit.^[24]^ The sensitivity and the specificity of this threshold were calculated.

### 2.7 Statistical analyses

Characteristics of patients were described by their frequency and percentages for categorical variables and by means and SDs or medians and interquartile ranges for quantitative variables.

Percentages were compared using the chi-square test or Fisher's exact test, as appropriate. Quantitative variables were compared using Student's *t* test or the Mann–Whitney test when the distribution was non-Gaussian.

The amount of missing data in this study was low. Analyses were performed on complete cases and no assumptions were made for the patterns of missing data. All statistical tests were performed at a 2-tailed type I error of 5%. All analyses were performed using R Statistical software, version 3.5.3.

## 3 Results

### 3.1 Validation study

Twelve centers (7 dermatologists and 5 rheumatologists) included at least one patient. One hundred seventeen patients participated in this study; all the patients fulfilled the inclusion and non-inclusion criteria. At baseline 97.4% of the patients (n = 114) completed the QSQ and 75 patients (64.1%) completed the day 7 QSQ. Among the 117 patients included, 95 (91.1%) were seen in clinics between month 3 and 6; among these 95 patients, 91 (95.8%) completed the QSQ between M3 and M6. The clinical and demographic characteristics of the patients are reported in Table 1.

**Table 1:** Demographic characteristics of the population participating in the psychometric validation of the QualipsoSex Questionnaire.

	Total N = 117
Age (years), mean (SD)	44.6 (12.7)
Male gender, n (%)	64 (54.7%)
BMI (kg/m^2^) mean (SD)	28.2 (6.0)
HAD depression (0–21)	7.0 (4.0–11.0)^∗^
Median (IQR)		
Mean (SD)	8.1 (5.0)
Fatigue NRS (0–10)	5.5 (3.0–7.0)^†^
Median (IQR)	
Mean (SD)	5.2 (2.5)

At baseline, 114 patients had Pso and 35 patients had PsA including 17 peripheral arthritis Psa, 4 had axial disease and 13 patients both axial disease and peripheral arthritis; one patient had an undifferentiated phenotype. In PsA, mean disease duration was 5 years in the patients with the peripheral arthritis phenotype (n = 30) and the mean BASDAI score was 6.5 in the patients with axial disease (n = 18). Specific PsA characteristics are reported in Table 2. Psoriasis mean disease duration was 18 years, the mean PASI score was 12.5/72 and the mean BSA involved was 20.9% in the 114 patients. Genital organs were involved in 44.7% of cases. Other specific Pso characteristics are reported in Table 3.

**Table 2:** Description of the 35 psoriatic arthritis patients included in the validation study.

	N-35
Disease duration (years)		
Median (IQR)	6.0 (2.0–11.0)
Mean (SD)	7.7 (6.3)
Subtypes, n (%)^∗^	
Peripheral disease	17 (50.0%)
Axial disease	4 (11.8%)
Peripheral disease + axial disease	13 (38.2%)
Peripheral arthritis (N = 30)	
Tender joint count^†^	
Median (IQR)	3.0 (1.0–6.0)
Mean (SD)	5.0 (9.8)
Swollen joint count^†^	
Median (IQR)	0.0 (0.0–1.0)
Mean (SD)	1.7 (3.3)
Localizations, n (%)	
Hand	16 (53.3%)
Elbow	2 (6.7%)
Shoulder	7 (23.3%)
Hip	3 (10.0%)
Knee	8 (26.7%)
Heel	7 (23.3%)
Foot	11 (36.7%)
Axial disease (N = 18)	
Score BASDAI^†^	
Median (IQR)	7.0 (5.7–8.1)
Mean (SD)	6.5 (2.3)
Localizations	
Cervical spine	9 (50.0%)
Thoracic spine	3 (16.7%)
Lumbar spine	11 (61.1%)
Sacroiliac	5 (27.8%)

**Table 3:** Specific psoriasis characteristics in the 114 psoriasis patients included in the validation study.

	N missing	N-114
Mean duration (years)	3	15.0 (8.0–25.0)
Median (IQR)–mean (SD)		18.1 (12.6)
PASI score (0–72)	1	12.0 (6.4–16.0)
Median (IQR)–mean (SD)		12.5 (8.9)
Body surface area (0–100%)	1	14.0 (7.0–30.0)
Median (IQR)–mean (SD)		20.9 (20.6)
Current affected area, n (%)		
Scalp	0	87/114 (76.3%)
Face	0	43/114 (37.7%)
Trunk	0	83/114 (72.8%)
Upper limbs	0	97/114 (85.1%)
Hands	0	60/114 (52.6%)
Nails	0	46/114 (40.3%)
Genitals	0	51/114 (44.7%)
Others	0	61/114 (53.5%)

At baseline 96/117 (82.8%) were treated including 56/96 (58.3%) treated with topical treatments and 24/96 (25%) with biologic therapies.

### 3.2 Psychological properties of the QSQ

Out of the 117 patients included, the number of usable QSQ (i.e., with only two missing answers) was 112 for the QSQ at baseline, 71 for the day 7 QSQ and 85 for the M3 to M6 QSQ.

### 3.3 Internal consistency validity

Internal consistency was excellent with a Cronbach's α of 0.87 (CI 95% 0.83–0.90).

Each question on the QSQ had a moderate to strong correlation with the total, except for Q8 which had a weak correlation with the total. Table 4 reports the Cronbach's α coefficients for each question on the QSQ.

**Table 4:** Cronbach's α coefficients for each question in the questionnaire.

Questions	Correlation with the total	Cronbach's α without the variable
Q1	0.65	0.86
Q2	0.76	0.85
Q3	0.73	0.85
Q4	0.69	0.85
Q5	0.51	0.87
Q6	0.53	0.86
Q7	0.57	0.86
Q8	0.37	0.88
Q9	0.55	0.86
Q10	0.57	0.86

### 3.4 Construct validity

The construct validity of the QSQ was assessed in 114 patients who filled in the baseline QSQ using an exploratory factorial analysis. Kaiser's “Measure of Sampling Adequacy” (MSA) showed a good adequacy to the factorial analysis model with an MSA = 0.84. The MSA index measures the factorial analysis model adequacy to the sample data; an MSA value above 0.80 was considered as ideal by Kaiser.^[25]^

### 3.5 Reliability

The reliability was assessed by the ICC in 35 patients who all considered that there had been “no change” in the overall assessment of change in their disease between baseline and day seven. The ICC was 0.93 (95% CI: 0.88–0.98). In these 35 patients the mean QSQ value was 16.1 ± 9.2 at baseline vs 15.4 ± 10.6 at day 7.

### 3.6 Criterion validity

The criterion validity was estimated using the Pearson correlation coefficient between the QSQ score and question 9 of the adapted DLQI in the 112 patients who completed the baseline QSQ. The correlation between the two scales was moderate with a Pearson correlation coefficient of 0.48 (95% CI: 0.32–0.61).

### 3.7 Responsiveness

The sensitivity to change was assessed in 57 patients who declared an improvement in the global assessment of their disease at M3 to M6 vs baseline and whose QSQ scores could be calculated at baseline and at M3 to M6. The effect size (standardized difference) was −0.86 (95% CI: −1.27, −0.46) in this sample. In those patients the mean QSQ score was 16.6 ± 9.9 at baseline and 8.1 ± 9.7 at M3 to M6.

### 3.8 Interpreting the QSQ

The PASS was assessed in 50 patients who declared themselves satisfied with their current status (i.e., who answered Yes to the question “If your sexual quality of life remains unchanged, would you consider it satisfactory?”). The PASS score (defined by the third quartile of the QSQ in these patients) could be assessed in 47 (94.0%) of them: one baseline score was not usable and two patients did not complete the QSQ at baseline. In this sample of patients, the PASS score was estimated at 18. At baseline, 58 patients out of 112 (51.8%) had a QSQ score ≤18. This preliminary cut-off value provided a sensitivity of 76.6% (95% CI: 62.0–87.7) and a specificity of 66.1% (95% CI: 53.3–77.4).

The MCID was assessed by the mean score difference in the QSQ value at M3 to M6 vs baseline in patients who claimed an improvement in their sexual quality of life (n = 37) as compared to patients who claimed no improvement in their sexual quality of life (n = 41).

In this sample, the MCID was found to be −7. This preliminary cut-off value provided a sensitivity of 56.8% (95% CI: 39.5–72.9) and a specificity of 73.2% (95% CI: 57.8–86.1).

## 4 Discussion

Both Pso and PsA can lead to sexual impairment.^[5,6,13,26,27]^ Sexual problems are frequently observed in Pso patients with sometimes a major impact on psychological parameters such as depression, self-esteem and negative image and self-worth.^[13]^ When hand, wrist, lower back or sacroiliac joint are involved, PsA is also known to interfere with the physical ability to have intimate relationships.^[5,26,28]^ Although many tools are available to measure certain aspects of sexual dysfunctions such as the International Index of Erectile Function or the Index of Female Sexual Function, there is no specific questionnaire that might help to understand the specific feelings in terms of sexual health of patients who suffer from Pso and PsA.

The present study aimed at checking the psychometric properties of the QSQ that assessed the perceived sexual quality of life in patients with Pso with cutaneous and/or rheumatic involvement. The QSQ comprises 10 questions rated from 0 to 4 with a total score ranging from 0 (no impact) to 40 (maximum impact).

The QSQ was found to have satisfactory psychometric properties. This questionnaire, developed with the participation of patient research partners, should enable better cross-talk between patients and medical doctors on a subject that might be considered as sensitive or intrusive in a patient's life.

The face and the content validity of the QSQ were established by the development of the questionnaire through the literature review, patient and physician interviews and the qualitative steps of the questionnaire development as described previously in.^[13]^ The internal consistency in this sample was considered as high (Cronbach's α coefficient: 0.87).^[22]^ Q8 resulted in the lowest (0.37) correlation with the total (see Table 4). Q8 is probably the question that focuses the most specifically on the impact of PsA on sexuality. Consequently, Pso patients without PsA will likely express lower values than PsA patients on this question. However, Pso patients may also suffer from osteoarthritis and express their pain and stiffness related to other musculoskeletal comorbidities than PsA^[29]^ The QSQ also demonstrated an adequate test–retest stability with an ICC of 0.93. According to the literature, this reliability can be considered excellent as it exceeds the threshold of 0.90.^[30,31]^ This result is consistent with the result obtained for another questionnaire (QualiSex) dealing with the impact of Rheumatoid Arthritis on sexuality, where the ICC was 0.83.^[32]^

Assessing the criterion validity, the Pearson correlation coefficient was 0.48 indicating a moderate correlation between the QSQ and question 9 on the DLQI. However, it can be argued that this might be linked to the lack of validity of question 9 on the DLQI.^[17]^

We also found that the QSQ had an effect size (standardized difference) of −0.86, confirming the sensitivity to change of the QSQ. According to the classification of Cohen^[33]^ this effect size can be considered as good.

The MCID was estimated at 7 points for improvement of quality of life. The low sensitivity value (56.8% [95% CI: 39.5–72.9]), meaning that with a reduction of 7 points a large proportion of patients may perceive improvements without reaching this cut-off value on QSQ, is probably due to the complexity of assessing sexual quality of life. The specificity level of 73.2% (95% CI: 57.8–86.1) is good. Further studies are needed to explore this value.

This study has both weaknesses and strengths. The strengths include the participation of patients’ partners in the elaboration of the qualitative steps of the QSQ, permitting the consideration of dimensions of sexual health that might be important from the patients’ perspective. The QSQ has good psychometric properties which were assessed using the appropriate methodology fulfilling the COSMIN checklist.^[21]^ The QSQ has potentially good generalizability. The QSQ was validated with 49 patients in the qualitative steps^[13]^ and the psychological quantitative steps in the present study included 114 patients.

Patients with PsA fulfilled the CASPAR classification criteria^[18]^; 25% of the patients were taking biological agents. Patients with current genital lesions exhibited significantly more sexual health problems as compared to patients not currently affected.^[6,27]^ In our sample of patients, 44.7% exhibited genital Pso lesions. Although this frequent localisation of Pso is recognized by patients, it is not expressed spontaneously to their physicians.^[34]^ Contrasting with physicians’ reluctance to tackle the subject of patients’ sexual health problems, it appears that there is a high level of acceptance among patients to consider questions of sexuality and intimacy.^[35]^ One can surmise that the more patients have difficulties in expressing their problems to the physician, the more sexual health issues are likely to be important.

In terms of limitations, patients were recruited from secondary or tertiary care referral centers. The QSQ could benefit from further validation in another setting such as community clinics. Limitations also include a potential cultural bias, since the items of the QSQ were obtained from a qualitative study of French patients and the expression of intimacy and sexuality might be different from one country to another. Furthermore, the QSQ serves only as a tool to explore and facilitate the dialogue on sexual health issues in Pso and PsA but does not substitute for a thorough examination by other health professionals such as sexologists and psychologists.

The QSQ questionnaire is presented in Figure S1; see in the Appendix, Supplemental Content.

The present study is the first attempt at developing a new patient-reported outcome measure to evaluate the perceived impact of Pso and PsA on a patient's sexuality. The QSQ is short (10 items), has demonstrated good psychometric properties fulfilling the validation process relative to the recommendations of the COSMIN check list.^[21]^ The QSQ is simple to score and may hopefully be valuable in clinical practice but also, thanks to its responsiveness, can be included in clinical trial protocols to measure the impact of specific interventions on sexuality in both Pso and PsA patients.

## Acknowledgments

The authors thank the patients and the association (France Psoriasis) who took part in this study. The authors also acknowledge Nukleus for methodological help in developing the wording and content of the questionnaire. The authors acknowledge Mrs Elisabeth Rowley for editorial assistance in preparing this manuscript for publication. The authors also thank Najat Gouyette MSD, Laurent Hertau UCB Pharma France, and Jean-Baptiste Quiniou Celgene. This study is part of the French network of University Hospitals HUGO. The QualipsoSex Questionnaire is protected by international copyright (CC-by), with all rights reserved E.L and E.E. For information on the QualipsoSex Questionnaire, please contact eric.lespessailles@chr-orleans.fr or eric.esteve@chr-orleans.fr.

## Author contributions

**Conceptualization:** Eric Lespessailles, François Maccari, Bénédicte Charles, Marc Marty, Eric Esteve.

**Data curation:** Eric Lespessailles, Marc Marty, Eric Esteve.

**Formal analysis:** Eric Lespessailles, Cyril Ferdynus, Marc Marty, Eric Esteve.

**Investigation:** Eric Lespessailles, Emmanuel Mahé, Ziad Reguiai, Edouard Begon, François Maccari, Nathalie Beneton, Guillaume Chaby, Carole Rosenberg, Emmanuelle Dernis, Fabienne Roux, Isabelle Henry-Desailly, Marc Marty, Eric Esteve.

**Methodology:** Eric Lespessailles, Cyril Ferdynus, Marc Marty, Eric Esteve.

**Supervision:** Eric Lespessailles, Marc Marty, Eric Esteve.

**Validation:** Eric Lespessailles, Cyril Ferdynus, Marc Marty, Eric Esteve.

**Writing – original draft:** Eric Lespessailles, Marc Marty, Eric Esteve.

**Writing – review & editing:** Eric Lespessailles, Emmanuel Mahé, Ziad Reguiai, Edouard Begon, François Maccari, Nathalie Beneton, Guillaume Chaby, Carole Rosenberg, Emmanuelle Dernis, Fabienne Roux, Isabelle Henry-Desailly, Bénédicte Charles, Cyril Ferdynus, Marc Marty, Eric Esteve.

## Supplementary Material

Supplemental Digital Content
